# Chiral visible light metasurface patterned in monocrystalline silicon by focused ion beam

**DOI:** 10.1038/s41598-018-29977-4

**Published:** 2018-08-02

**Authors:** Maxim V. Gorkunov, Oleg Y. Rogov, Alexey V. Kondratov, Vladimir V. Artemov, Radmir V. Gainutdinov, Alexander A. Ezhov

**Affiliations:** 1 Shubnikov Institute of Crystallography, Federal Scientific Research Centre “Crystallography and Photonics”, Russian Academy of Sciences, Moscow, 119333 Russia; 2 National Research Nuclear University MEPhI (Moscow Engineering Physics Institute), Moscow, 115409 Russia; 3 Faculty of Physics, Lomonosov Moscow State University, Moscow, 119991 Russia; 4 Topchiev Institute of Petrochemical Synthesis, Russian Academy of Science, Moscow, 119991 Russia

## Abstract

High refractive index makes silicon the optimal platform for dielectric metasurfaces capable of versatile control of light. Among various silicon modifications, its monocrystalline form has the weakest visible light absorption but requires a careful choice of the fabrication technique to avoid damage, contamination or amorphization. Presently prevailing chemical etching can shape thin silicon layers into two-dimensional patterns consisting of strips and posts with vertical walls and equal height. Here, the possibility to create silicon nanostructure of truly tree-dimensional shape by means of the focused ion beam lithography is explored, and a 300 nm thin film of monocrystalline epitaxial silicon on sapphire is patterned with a chiral nanoscale relief. It is demonstrated that exposing silicon to the ion beam causes a substantial drop of the visible transparency, which, however, is completely restored by annealing with oxidation of the damaged surface layer. As a result, the fabricated chiral metasurface combines high (50–80%) transmittance with the circular dichroism of up to 0.5 and the optical activity of up to 20° in the visible range. Being also remarkably durable, it possesses crystal-grade hardness, heat resistance up to 1000 °C and the inertness of glass.

## Introduction

Dielectric metasurfaces exhibit an impressive combination of functional optical properties^1,2^ including resonant electric and magnetic responses^3^ and high optical chirality^4^, and are capable of efficient control of the light phase, polarization and propagation direction within fractions of the free space wavelength^5,6^.

Similarly to plasmonic metallic metasurfaces^7,8^, the dielectric ones host optical resonances, which emerge as standing electromagnetic waves confined within the dielectric elements. For a metasurface to be non-diffracting, its unit cell has to be subwavelength with respect to the free space light, and, therefore, the dielectric resonators should possess large refractive index. Their shape and size determine the optical performance while the functional efficiency in the most applications requires the dielectric absorption loss to be low.

Among the variety of suitable materials with high refractive index in the visible and near infrared ranges, silicon is the most explored one and promises the fastest integration of the metasurfaces into the existing technological routes. In the visible range, the monocrystalline form of silicon (mono-c-Si) has the highest transparency^6,9^, while the other modifications (polycrystalline poly-c-Si and, especially, amorphous a-Si) absorb light substantially stronger^10^. In the near infrared range, where the absorption loss in poly-c-Si is moderate, one can also efficiently use it as a platform for metasurfaces capable of multifarious light transformations^11–14^. Convenience of sputtering thin poly-c-Si layers upon arbitrary optical substrates explains why it has been also used in the early^15^ as well as in the recent^5,16^ experiments in the visible range.

In the optimal case, however, a visible light silicon metasurface has to be shaped from a mono-c-Si layer on a transparent substrate of lower refractive index. As most commercially available silicon-on-insulator (SOI) wafers designed for electronic applications are supported by thick silicon slabs, one has to perform extra preparation steps to obtain, e.g., a thin mono-c-Si layer glued to glass^6,17^. In this regard, valuable simplicity is offered by the silicon on sapphire (SOS) platform with a thin mono-c-Si layer epitaxially grown on the R-cut surface of sapphire crystal. The latter is perfectly transparent in broad optical and infrared ranges and forms an extremely durable structure with the silicon.

For a mono-c-Si metasurface fabrication, the choice of patterning technique is critically important, as it can easily generate structural defects and convert mono-c-Si into poly-c-Si or even a-Si, which boosts the optical absorption loss to inappropriately high levels^18,19^. The electron beam lithography followed by the chemical etching is widely employed as it can produce regular vertical grooves and wells in mono-c-Si films without noticeably affecting the quality of the remaining material. However, the technique is incapable of creating complex submicrometer shapes and presently known more sophisticated etched structures have a size of at least a few micrometers^20^. Accordingly, the most common lithographically cut optical silicon metasurfaces consist of nanoridges^6^, nanorods^12^, nanodisks^16,21^, nanopillars^11,22^, nanoposts^5^, nanofins^23^, etc., which are all effectively two-dimensional shapes with vertical walls and height bound to be equal to the thickness of the initial mono-c-Si layer. As an alternative, the simplest 3D shapes–nanospheres–consisting of poly-c-Si have been produced and then converted into the mono-c-Si form by subsequent femtosecond laser pulse irradiation^24^. Recently, poly-c-Si nanoparticles of more complex chiral crescent shapes have been fabricated by means of the gradient mask transfer technique^25^.

Since its early days, the focused ion beam (FIB) lithography has been recognized as a convenient tool for nanofabrication of emerging optical materials^26,27^. The technique produces minor effect on the optical response of metals dominated by the conduction electrons, and has been applied for shaping the metallic parts of numerous plasmonic metasurfaces and metadevices^28–30^. Current dual beam systems of the electron-ion microscopy allow fast processing of relatively large areas. The advanced options of controlling the FIB scanning sequence by programmable digital templates enable fabrication of arrays with truly three-dimensional (3D) unit cell shapes^31–33^.

Being technically capable of versatile silicon patterning^34^, the FIB lithography is known to produce 30–50 nm thick damaged layers^35^. The related significant increase of the optical absorption loss^18^ has prevented the technique from being applied to the fabrication of Si-based optical structures. Although some early works suggested the possibility to restore the functionality of FIB-exposed silicon waveguides by annealing^19^, the particular prospects remained unexplored for a decade. Our recent electron microscopy studies have confirmed that FIB patterning of a SOS film generates a 50 nm thick damaged surface layer, which is also heavily contaminated with implanted gallium atoms^36^. Optical transmission measurements confirmed this layer to have an extremely adverse effect on the optical properties. High temperature annealing with oxidation was found to transform it into an oxide glass-like coating and to restore the transparency. Intriguing indications of polarizing features of the patterned SOS layer otherwise overshadowed by the strong light absorption were observed.

In this paper, we perform a comprehensive study of the optically functional mono-c-Si based complex shaped dielectric metasurfaces produced by fast and precise FIB patterning followed by appropriate high temperature annealing. To illustrate the merit of the advance, we fabricate a chiral 4-fold rotationally symmetric mono-c-Si metasurface. The optical chirality critically depends on the lack of mirror symmetry planes^37^, and although formally the symmetry of planar 2D structures can be broken by the presence of substrates^38,39^, in optical experiments such structures are clearly outperformed by those of proper 3D-chiral shape^40^. As the widely accepted chemical etching is restricted to producing flat Si blocks, the chiral Si-based metasurfaces known so far rely on the symmetry breaking by the substrate. Due to the lack of rotational symmetry axes such metasurfaces also do not possess circularly polarized optical eigenmodes^12,41^. We demonstrate that applying the FIB technique provides a valuable opportunity to handle sophisticated 3D shapes and to create chiral silicon metasurfaces free of such disadvantages and combining high transparency with strong optical chirality and notable durability. We describe the fabrication routine and precisely reconstruct the final composition of the unit cell. We perform comprehensive optical studies and show that they are in-line with the results of full-scale electromagnetic simulations. We also formulate a simple semi-phenomenological model that points at the chiral guided-mode resonance as the origin of the strong optical chirality.

## Results and Discussion

### Fabrication and structure reconstruction

Symmetry aspects are of key importance for the design of chiral metasurfaces. While the chirality by itself requires the absence of mirror symmetry planes, rotational axes of the order of 3 and higher ensure that circularly polarized waves are the true eigenmodes and all polarization transformations during the transmission of normally incident light are caused by the metasurface chirality^42^.

**Figure 1:**
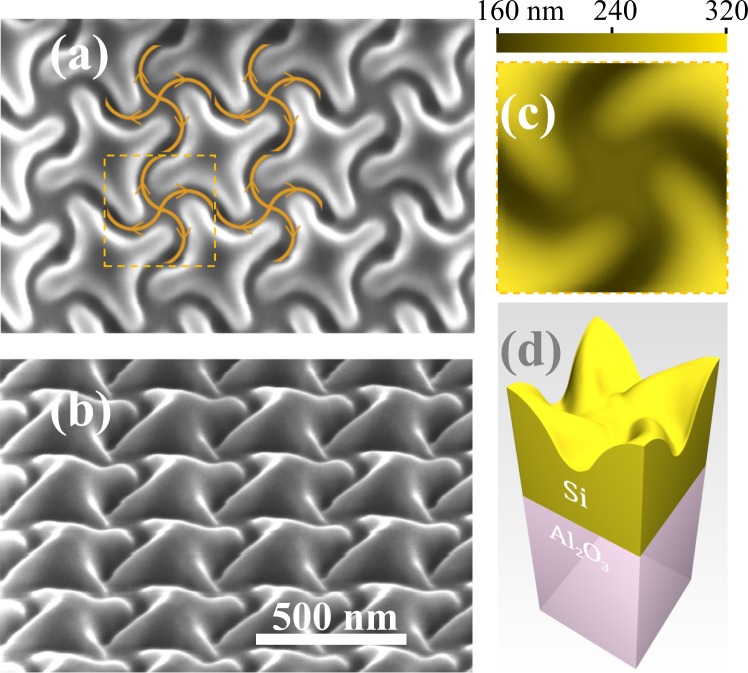
Chiral patterning of SOS produced by digitally controlled FIB: (**a**) SEM-image, normal view, with the FIB paths and directions (curved arrows) and the square unit cell (dashed boundary); (**b**) SEM-image of the sample tilted by 52°; (**c**) average surface relief within a unit cell resolved by AFM; (**d**) 3D-model of the average unit cell.

Accordingly, we select a 4-fold symmetric square lattice of twisted crosses, which is shown in Fig. 1(a). In order to avoid the light diffraction in the visible range, the lattice period is set to 370 nm. This way, circular areas of 68 *μ*m in diameter are patterned by the FIB (see Methods). The samples differently oriented with respect to the sapphire substrate crystallographic axes are fabricated along with a few test samples of smaller area for the reconstruction purposes. As seen in Fig. 1(b), the patterning results in a smooth complex-shaped relief of the mono-c-Si surface. Its precise reconstruction (see Methods) by means of the atomic force microscopy (AFM) reveals the averaged unit cell relief map presented in Fig. 1(c), where the colour specifies the height as measured from the flat Si/Al_2_O_3_ interface. The corresponding digital 3D model of the patterned SOS presented in Fig. 1(d) shows that the FIB creates 150 nm deep and ∼100 nm wide curved grooves. Note that the maximum SOS height exceeds the initial 300 nm thickness of the SOS layer, which indicates a substantial overdeposition of silicon from the grooves onto the adjacent regions unexposed to the FIB.

It has been established that shaping mono-c-Si with FIB damages its structure and creates layers which strongly absorb visible light^18^. The optical studies of the fabricated metasurface presented below confirm this general rule. Our recent transmission electron microscopy studies^36^ have revealed that processing with FIB gives rise to a  50 nm thick layer of damaged silicon, where the crystalline structure is altered and Ga atoms are implanted.

**Figure 2:**
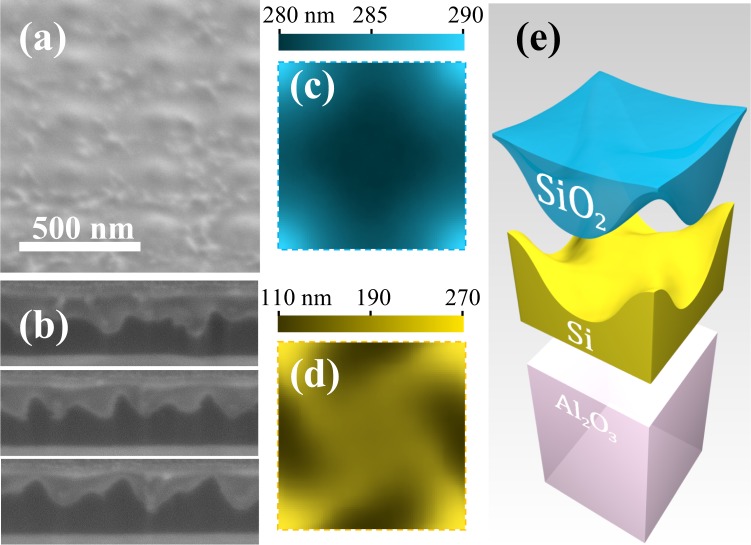
Chiral metasurface upon annealing: SEM-image of the surface, tilted by 52° view (**a**); three exemplary consecutive FIB cuts used for the 3D reconstruction (**b**); average surface relief within a unit cell resolved by AFM (**c**); profile of the Si/SiO_2_ interface resolved by 3D reconstruction (**d**); 3D model of the unit cell after annealing (**e**).

In order to restore the transparency in the visible range, the samples are subjected to the annealing with surface thermal oxidation (see Methods). This produces significant changes to the surface relief, as is illustrated by the typical SEM view shown in Fig. 2(a), where the complex chiral pattern is unobservable beneath the almost flat top SiO_2_ surface. AFM studies reveal a relief with a root mean square roughness of 0.5 nm and in-plane correlation lengths in the range between 15 and 20 nm. On the larger scale, the surface has a weak periodic modulation and its average unit cell map of heights measured from the Si/Al_2_O_3_ interface is shown in Fig. 2(c).

At the same time, sequences of FIB cross sections of the test samples (see Fig. 2(b) reveal that the Si/SiO_2_ interface underneath the SiO_2_ layer has a truly chiral complex shape. By means of the FIB 3D reconstruction we resolve this shape and create the average unit cell map of heights measured from the flat Si/Al_2_O_3_ interface presented in Fig. 2(d). Combining all the microscopic data, we produce the overall 3D model of the three-layered structure of the annealed chiral metasurface shown in Fig. 2(e). Apparently, the oxidation consumes a substantial part of the patterned SOS layer, but the remaining mono-c-Si retains a pronounced shape chirality.

### Optical properties

**Figure 3:**
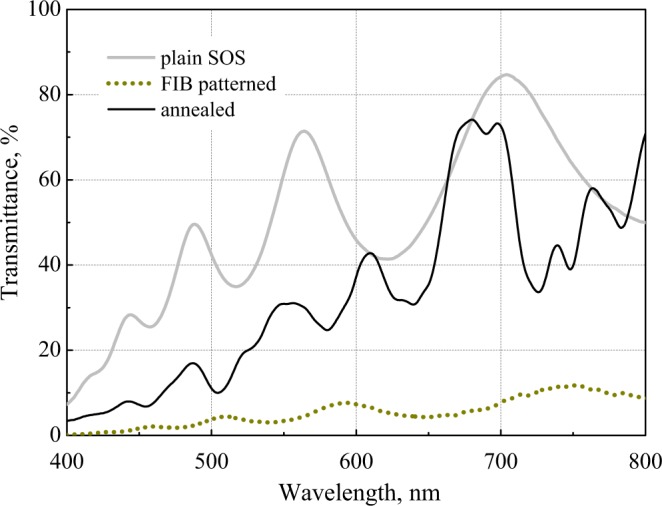
Spectra of the transmittance of linearly polarized light by the plain 300 nm SOS layer, metasurface after FIB-patterning, and upon the annealing with oxidation.

Microspectrometric transmission measurements (see Methods) demonstrate the efficiency of the annealing with oxidation for restoring the metasurface transparency in the visible range. As shown in Fig. 3, the unprocessed plain SOS transmission spectrum has the classical Fabry-Perot oscillations that indicate the homogeneity and transparency of the epitaxial mono-c-Si layer. After the FIB patterning, the same measurements reveal a dramatic increase of the light absorption as the transmission decreases by an order of magnitude. Upon annealing, the overall level of transparency is restored, while the transmission spectrum apparently differs from that of the initial plain SOS layer.

From the symmetry point of view, for the normally incident light, the optical properties of a chiral 4-fold rotationally symmetric metasurface are identical to those of a layer of isotropic chiral medium^42^. The corresponding key optical chirality parameters–circular dichroism (CD) and optical activity (OA)–can be resolved by different methods, and are independent of the sample orientation with respect to the incident linear polarization as well as, due to the Lorentz reciprocity, of the side of incidence^37^. Therefore, in order to confirm that the annealed metasurface retains the translational and rotational symmetries and to unambiguously characterize its chirality, we combine two different optical techniques. In the first method, hereinafter denoted as transmission ellipsometry, OA-spectropolarimetry and CD-spectrophotometry of the transmitted light are simultaneously performed (see Methods). Preliminary, for the linearly polarized light incident normally from the sapphire substrate, a pair of mutually orthogonal polarization directions are identified as those preserved during the transmission through the unprocessed SOS areas. Next, for those linear polarizations, the polarization state of the light transmitted through the metasurfaces is analysed. In this approach, the metasurface OA is defined as the angle of the relative rotation of the main axis of the transmitted polarization ellipse from the incident linear polarization direction. The ellipticity *e* of the transmitted light polarization characterizes CD of the metasurface as *CD* = sin2*e*.

In the second method, which we call CD-spectrometry, the left circularly polarized (LCP) and right circularly polarized (RCP) transmittances, *T*_*L*_ and *T*_*R*_ correspondingly, are resolved by means of microspectrometric measurements with the circularly polarized light incident directly onto the metasurface samples from the air. In this technique, CD is evaluated as *CD* = (*T*_R_ − *T*_L_)/(*T*_R_ + *T*_L_).

**Figure 4:**
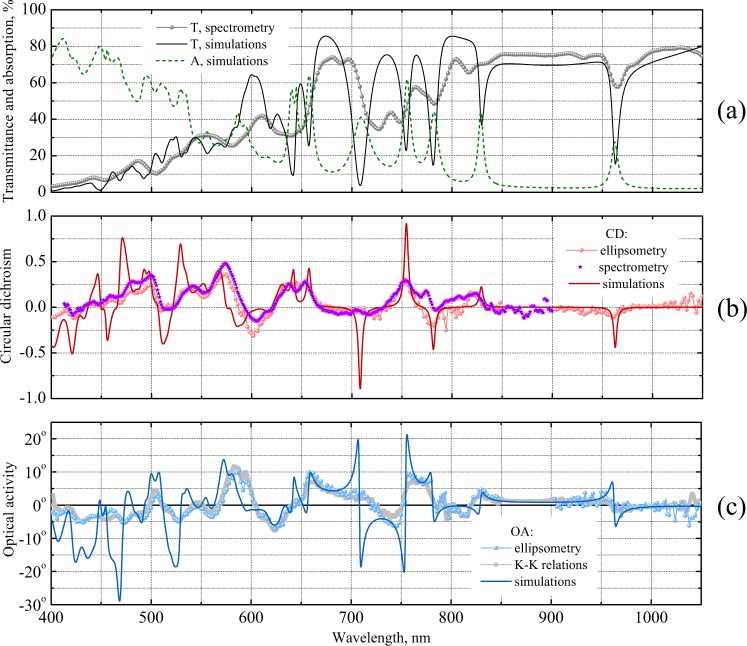
Numerically obtained (solid and dashed lines) and experimentally observed (scattered points) spectra of the optical properties of chiral metasurface: absorption (A) and transmittance (T) of linearly polarized light resolved by microspectrometry (**a**); CD resolved by transmission ellipsometry and microspectrometry (**b**); OA resolved by transmission ellipsometry and evaluated using Kramers-Kronig relations from the CD spectrum measured by transmission ellipsometry (**c**).

Generally, we observe an encouraging consistency of the CD and OA spectra measured for differently oriented samples and using different incident polarizations. Averaging the optical data over the samples and polarizations allows to improve the ellipsometry data quality in a broad wavelength range. Comparison of the CD spectra resolved by means of the ellipsometry and spectrometry in Fig. 4(b) demonstrates a remarkable agreement between the results of the two completely different measurement techniques. Spectrum of the OA obtained by the ellipsometry shown in Fig. 4(c) exhibits higher noise level at longer wavelengths due to worse spectral efficiency of the ellipsometer spectrometer resulting in lower consistency of the spectra of different samples and for different incident polarizations. For a final check, we employ the Kramers-Kronig relations between OA and CD. Being well known for natural weakly chiral materials^37,43^, the relations can be generalized for strongly chiral metasurfaces^32^. Accordingly, we evaluate the spectrum of OA from the broadband spectrum of CD measured by the transmission ellipsometry. As seen in Fig. 3(c), the obtained dataset accurately follows the experimental OA values. Altogether, the thorough analysis of the collected optical data unambiguously proves that the metasurface samples retain their rotationally symmetry and combine strong optical chirality with high transparency.

### Numerical simulations and discussion

For the full-scale electromagnetic modelling (see Methods), we take the metasurface unit cell obtained by combining the microscopic data (see Fig. 2(e)) and assuming the tabulated permittivity of mono-c-Si^44^, *ε* = 2.25 of the SiO_2_ layer, and *ε* = 3.1 of the sapphire substrate.

As shown in Fig. 4(a), the simulations quantitatively well reproduce the overall trend of the metasurface transmittance in a very broad wavelength range. Importantly, the simulated transmittance is very sensitive to the permittivity values assigned to Si within the multilayered structure shown in Fig. 2(e). For example, taking exactly the same structure but with the elevated silicon parts consisting of amorphous a-Si^10^ lowers the simulated transmittance by several times everywhere below the 800 nm wavelength. Therefore, the consistence of the observed and simulated data indicates a good optical quality of the silicon remaining after the oxidation.

The calculated transmittance spectrum experiences a number of sharp dips down to rather low values, some of which (see, e.g., the vicinity of 965 nm wavelength) are resolved by the experiment, although as considerably smoother and shallower ones. Sharper transmission dips are either observed as merged into a broader single one (as those at 642 and 656 nm wavelengths) or remain unobserved (e.g. as the one at 830 nm). According to the simulated absorption spectrum, the transmission dips are accompanied by sharp resonant increase of the losses which otherwise stay on the background level below 10% in the red and near infrared ranges.

Comparison of the simulated and measured optical chirality parameters in Fig. 4(b) and (c) indicates the overall adequacy of our numerical model, which reproduces most of the complex features of the OA and CD spectra. One can see that the simulated CD spectrum has a number of sharp resonances at the wavelengths exactly corresponding to the transmission dips. The simulated OA spectrum exhibits sharp antiresonances at those wavelengths. In the experiment, some of the sharp features are observed as smoother CD resonances and OA antiresonances (see e.g. the vicinity of 755 nm wavelength), while other more acute features remain unresolved (see e.g. the range around 710 nm).

**Figure 5:**
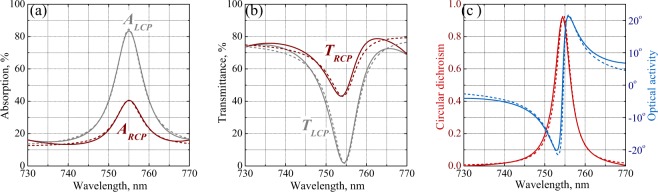
Simulated resonance of the optical properties of chiral metasurface (solid) and their fit with single resonance chiral COM theory (dashed): absorption (**a**) and transmittance (**b**) of LCP and RCP light; CD and OA spectra (**c**). The COM theory parameters are: *ω*_0_ = 2.49 ⋅ 10^15^ rad/s (which corresponds to *λ*_0_ = 755 nm), *γ* = 1.31 ⋅ 10^13^ rad/s, *α*_0_ = 0.119, *α*_R_ = 0.287, *α*_L_ = 0.727, *τ*_0_ = 0.882, *τ*_R_ = 0.235 *e*^2.78*i*^ and *τ*_L_ = 0.763 *e*^3.03*i*^.

Sharp spectral anomalies indicate the presence of high quality factor resonances which are generally typical for dielectric metasurfaces. In a metasurface consisting of patterned layer of highly refracting transparent material, such resonances appear in the form of the so-called guided-mode resonances, which occur when the incident plane waves diffract into the modes guided by the layer. Some years ago, it was shown how a periodic chiral patterning of a TiO_2_ layer gives rise to very similar narrow CD resonances and OA antiresonances^45^. In our case, this occurs in a layer of mono-c-Si with a 1.5 times higher refractive index which makes the grid of resonances noticeably denser. At shorter wavelengths, this causes their merging into a hardly comprehensible grid of peaks and dips on the spectra of optical observables. At longer wavelengths, one can isolate and analyze a single resonance, as is illustrated in Fig. 5 by a detailed view of the simulated absorption peak, transmission dip, CD resonance and OA antiresonance around 755 nm wavelength also observed by the experiments.

One can formulate a relatively simple analytical model describing the emergence of optical chirality due to a guided-mode resonance similar to the recent chiral coupled mode (COM) model developed for metallic nanostructures with extreme optical chirality due to multiple plasmon resonances^42^. Within COM models, the resonances are accounted independently of their particular nature. The model equations phenomenologically describe the excitation of the resonances by incoming plane waves and their irradiation into outgoing plane waves. Being similar to the conventional scalar COM model^46^, the chiral version takes into account the polarization effects responsible for the optical chirality: one introduces conjugate pairs of resonances of different handedness which interact with the incoming and outgoing plane waves of circular polarizations of the same handedness.

Although general formulation of the chiral COM model implies introduction of many phenomenological parameters, their number can be substantially reduced by applying the basic principles of symmetry and reciprocity. In particular, the reciprocity enforces a degeneracy of the conjugated resonances which are to possess equal frequencies and half-widths^42^. Note that it is even easier to apply this methodology to the chiral silicon metasurface, as its resonances possess higher quality factors and one can neglect their coupling.

Accordingly, for an isolated resonance as in Fig. 5, we apply a reduced single-resonant version of the formalism developed for plasmonic metasurfaces^42^. The solution of the COM model equations yields Lorentzian dispersion of the absorption:

1 $${A}_{{\rm{R}},{\rm{L}}}^{^{\prime} ,^{\prime\prime} }={\alpha }_{0}+\frac{{\alpha }_{{\rm{R}},L}^{^{\prime} ,^{\prime\prime} }}{{({\omega }_{0}-\omega )}^{2}+{\gamma }^{2}},$$

which is different for the RCP and LCP polarized incident waves (subscripts R and L) and the sides of incidence (superscripts ′ and ′′). The transmission amplitudes possess a Fano-type dispersion:

2 $${t}_{{\rm{R}},{\rm{L}}}(\omega )={\tau }_{0}+\frac{\gamma {\tau }_{{\rm{R}},L}}{i({\omega }_{0}-\omega )+\gamma }$$

and are independent of the side of incidence in accordance with the Lorentz reciprocity. Here the parameters *α*_0_ and *τ*_0_ describe the direct achiral non-resonant transmission and absorption correspondingly, *ω*_0_ is the resonance frequency and *γ* is its half-width. The partial absorption and transmission amplitudes, ${\alpha }_{{\rm{R}},L}^{^{\prime} ,^{\prime\prime} }$ and *τ*_R,L_, are determined by the parameters of the coupling of the conjugate resonances to the free-space circularly polarized plane waves. The metasurface optical chirality is then explained as a result of the chirality of this coupling.

The observable transmission characteristics expressed as *T*_R,L_ = |*t*_R,L_|^2^, $OA=\frac{1}{2}({\rm{\arg }}\,{t}_{{\rm{L}}}-{\rm{\arg }}\,{t}_{{\rm{R}}})$, and *CD* = (|*t*_R_|^2^ − |*t*_L_|^2^)/(|*t*_R_|^2^ + |*t*_L_|^2^) are all determined by a few model constants: the resonance frequency *ω*_0_ and half-width *γ*, and by the partial transmission amplitudes *τ*_0_ and *τ*_R,L_. Since only the difference between the phases of the complex amplitudes *t*_R_ and *t*_L_ is relevant, the observables are fully characterized by 7 real parameters. As illustrated in Fig. 5, this simple approach allows us to reproduce very precisely all the data in the vicinity of an isolated resonance obtained by the full-scale FDTD simulations.

In accordance with Eq. (1), the absorption peaks in Fig. 5(a) have the Lorentzian shape and their equal resonance frequency and half-width yield the resonance quality factor as high as ${\omega }_{0}/\gamma \simeq 190$. For comparison, this value exceeds by an order of magnitude that typical of plasmon resonances hosted by metallic chiral metasurfaces: the quality factors of the resonances of relatively transparent arrays of complex shaped particles^40^ as well as of more opaque arrays of chiral holes^42^ do not exceed 20.

Although narrower resonances with higher quality factors are predicted by the FDTD simulations, they remain unobserved experimentally. Such guided mode resonances rely on extremely weak coupling of the leaky guided modes with the free-space plane waves and require high degree of the metasurface translational order. In our case, the scattering loss caused by the structure imperfections overshadows the weak coupling and suppresses the narrower resonance excitation. We believe also that the scattering is generally responsible for the considerable widening and shallowing of all observed resonances compared to those in the simulated spectra.

As seen in Figs. 5(a), 3D-chiral patterning of the Si layer gives rise to a strong selectivity of the coupling of sharp resonances to the plane free-space circularly polarized waves. This allows achieving strong OA and CD in the range of relatively high transmittance. As the electromagnetic fields of optical resonances of dielectric nanostructures are generally confined in the parts with high refractive index, the shape of the Si layer is more important than its environment. To illustrate this, we simulate also the light transmission through a similarly arranged 2D-chiral silicon structure, in which the pattern shown in Fig. 1(a) consists of twisted crosses of 70 nm wide slits with vertical walls cut through a 150 nm thin SOS layer. From the formal symmetry point of view, the optical chirality of such structure appears only due to the mirror symmetry breaking by the presence of the sapphire substrate. Physically, the 2D-chiral Si blocks host sharp dielectric resonances, while the substrate perturbs them and provides a chiral selectivity to their coupling to the free-space light. This effect, however, appears to be very weak and we are able to achieve substantial optical chirality only upon strong resonant suppression of the transmission, i.e., with both LCP and RCP transmittances on the level of a few percent.

One should also note the key positive role of the regular light absorption in mono-c-Si in the build up of the optical chirality. It is known, that CD of rotationally symmetric metasurfaces appears solely due to the difference of the RCP and LCP light absorptions^42^. On the other hand, the generalized Kramers-Kronig relations implicate that the CD resonances are accompanied by proportionate OA antiresonances^32^. Therefore, in the range of weak light absorption in mono-c-Si in the near infrared range, even strong resonances producing pronounced impact on the observed transmittance do not result in substantial CD or OA (see, for instance, Fig. 4 in the vicinity of 965 nm wavelength).

The simulated spectra are a good reference point to discuss the optical capabilities of 3D chiral silicon metasurfaces. As illustrated by Fig. 5, one can realize an efficient circular polarizer transmitting about 45% of the RCP and only 1–2% of the LCP light in a narrow resonant range. Although the current structure lacks broadband chiral effects, its high quality factor resonances are remarkably similar to those of the chiral metallic metasurfaces in the microwave^47^ and terahertz^48^ ranges. There, it is possible to achieve broadband OA by exploiting specific combinations of the resonances that give rise to the so-called Blaschke contribution to the OA^32,48^. A chiral silicon metasurface with similarly arranged resonances will operate as a broadband omnidirectional polarization rotator for the visible light.

More generally, the fabricated metasurface illustrates the feasibility of creating arbitrarily shaped 3D silicon nanostructures for the visible range using the commonly available FIB laboratory equipment. While currently the theoretical design of silicon metasurfaces is primarily focused on the available lithographically cut structures, nanopatterned silicon layers are also getting attention and, for instance, recent simulations have shown that their guided mode resonances facilitate the nonreciprocity of the nonlinear transmission regime^49^.

## Conclusion

We show that FIB lithography followed by annealing with oxidation of the damaged layer can produce complex shaped mono-c-Si metasurfaces. As a particular example, we apply 4-fold symmetric chiral patterning to a mono-c-Si layer on sapphire and fabricate a metasurface, which combines high transparency with strong optical chirality. Light transmission experiments verify the metasurface rotational chiral symmetry and suggest that its performance is limited by the scattering on the imperfections arising during the annealing. Adjusting the fabrication routine to improve the translational order will allow achieving even higher levels of the optical chirality.

## Methods

### FIB patterning

FIB patterning of the commercial 300 nm epitaxial mono-c-Si films on monocrystalline sapphire are performed using FEI Scios DualBeam system. For the patterning, the beam of a current of 0.1 nA of Ga^+^ ions accelerated to an energy of 30 keV is used. The beam path is controlled by the digital templates which specify subsequent milling of square unit cells, and within each cell the FIB follows the twisted cross paths starting from the center outwards as illustrated in Fig. 1(a).

### Annealing with oxidation

Preceded by the sample cleaning with *ex-situ* plasma argon-oxygen mixture for one hour in Fischione 1070 NanoClean system, the annealing is carried out in a custom furnace for 30 minutes in dry air at 1100° C, which, according to the Massoud silicon oxidation model^50^, transforms the top 30–35 nm thick Si layer into $\simeq 75$ nm layer of SiO_2_.

### AFM surface reconstruction

Precise reconstruction of the relief is carried out on NT-MDT NTEGRA Prima system in the tapping mode using custom sharpened vertical probes. The measurements of the metasurface after FIB patterning include flat unprocessed SOS areas and the obtained height profiles are normalized with respect to them. Calibrated 300 nm thickness of the SOS layer is used to recalculate the heights from the bottom Si/Al_2_O_3_ interface. The height map of the top of the annealed metasurface is normalized according to the FIB 3D reconstruction data. All obtained AFM images are processed with a specific numerical routine that includes a subtraction of the tip curvature radius, noise reduction, and a two-step averaging over all unit cells and their 4-fold rotations^42,51^.

### FIB 3D reconstruction

Reconstruction of the oxidized annealed metasurface is preceded by deposition of a 200 nm thick Pt protection layer using the FEI Scios gas injection system. The initial 20 nm thick Pt layer was deposited by the electron-beam-induced deposition, while the rest is deposited using the much faster FIB-induced deposition. To perform the 3D reconstruction, 120 equidistant consecutive sections with a pitch of 20 nm and a depth of 1.5 *μ*m were milled with the FIB of a current of 0.1 nA of Ga^+^ ions accelerated to an energy of 30 keV. SEM images of each nanostructure cross-section are acquired using the T2 in-lens detector enhancing the contrast between the constituent materials. The images are then combined in a pool and post-processed with the FEI Avizo 9 software. The relative alignment of successive images is determined by the ordinary least squares algorithm. The material layer segmentation is performed using a gray-scale-based threshold filtering, which allows to preserve the small shape features. The obtained profiles of the interfaces between the layers are subjected to the same averaging routine^51^ as the AFM data above.

### Microspectrometry

Light transmission measurements are performed in a wide spectral range (400–1050 nm) using a setup with Olympus CX31-P trinocular polarized-light microscope equipped with fiber-optic Avantes AvaSpec-2048-USB2-UA spectrometer. Measurements are done with a 40x microobjective which allows to collect the light transmitted through a round region of the area about 30 *μ*m^2^. The microscope halogen lamp is used as a broadband unpolarized light source.

### Transmission ellipsometry

OA and CD are measured by using the linearly polarized normally incident light and characterizing the polarization state of the transmitted light with Horiba Jobin-Yvon UVISEL 2 spectroscopic ellipsometer. The obtained ellipsometric angles are recalculated by custom scripts to the polarimetric values corresponding to OA and CD. The used rectangular light spot of an area of 50 × 70 *μ*m^2^ is sufficiently small to study individual metasurface samples. The surface of the R-cut sapphire substrate forms an angle of 57.6° with the optical axis of the crystalline sapphire^52^, and the substrate birefringence also contributes to the measured polarizing properties. To identify the metasurface contribution and eliminate that of the substrate, two specific linear polarizations of light incident on the metasurface from the sapphire are used. They are chosen empirically so that no polarization transformation occurs during the transmission through the unprocessed SOS areas, which corresponds to the light polarized along or perpendicular to the projection of the sapphire optical axis on the R-cut surface.

### CD-spectrometry

CD spectrometry is performed using CLSM FV1000 confocal laser scanning microscope based on IX81 inverted microscope equipped with Olympus scanning unit with a spectral type detection system. External Schott ACE halogen light source is used with the internal IR interference filter removed to expand the spectrum. LCP and RCP light states incident from the air onto the metasurface are obtained by Moxtek UBB01A ultra-broadband wire-grid polarizer combined with achromatic Thorlabs AQWP05M-600 quarter-wave plate. 10x microobjective is used and the spatial resolution of at least of 3 *μ*m is regulated by the size of the confocal aperture. Spectra in the range 400–800 nm are recorded as CLSM raster and post-processed with CLSM software.

### Numerical modeling

Full-scale FDTD modeling is performed using commercial package Speag SEMCAD X v14.8 with Acceleware CUDA GPGPU acceleration library running on a high-performance workstation equipped with a pair of 10-core Intel Xeon processors and Nvidia Tesla K40 GPU. Periodic boundary conditions are applied to the side faces of the reconstructed unit cell (Fig. 2(e)), while the top and bottom faces of the simulation box are set to light absorbing perfectly matched layers. The simulated light plane wave is incident upon the patterned silicon surface from the vacuum. FDTD grid step of 4 nm is used as it has been checked that further decreasing the step does not affect the optical observables. Arrays of field monitors are placed above and below the metasurface to resolve the contributions from the incident, transmitted and diffracted waves. The absorption is evaluated as the deficit of the light energy between the incident and all the outgoing waves.
